# Ultrasonication modifies the structural, thermal and functional properties of pumpkin seed protein isolate (PSPI)

**DOI:** 10.1016/j.ultsonch.2024.107172

**Published:** 2024-11-22

**Authors:** Mehvish Habib, Sakshi Singh, Sameer Ahmad, Shumaila Jan, Ankit Gupta, Kulsum Jan, Khalid Bashir

**Affiliations:** a Department of Food Technology, Jamia Hamdard, New Delhi 110062, India; b Department of Food Science & Technology, NIFTEM-K, 131028, India; c Department of Molecular Medicine, Jamia Hamdard, New Delhi 110062, India

**Keywords:** Pumpkin seed protein isolate, Ultrasound, Physicochemical characteristic, Molecular changes

## Abstract

Protein isolates from pumpkin seeds were prepared and then treated with high-intensity ultrasound (HIUS) using a probe-based method. The impact of ultrasonication on the physicochemical, molecular, and thermal properties of these isolates were analyzed and compared to untreated controls. Results showed significant improvements (p ≤ 0.05) in color (L*, a*, b* values), solubility, emulsification capacity, and stability, as well as a reduction in molecular weight, indicating enhanced functionality of the pumpkin seed protein isolates (PSPIs) after HIUS treatment. However, HIUS treatment decreased the denaturation temperature (Td), denaturation enthalpy (ΔH), thermal stability, and particle size of the isolates. With treatment durations ranging from 5 to 20 min, T_d_ dropped from 67.31 °C to 56.38 °C, and ΔH declined from 45.78 to 35.43 J/g, likely due to structural and conformational modifications from ultrasonic-induced molecular bond disruptions. The greatest reduction in particle size, from 117.46 μm to 85.26 μm, was observed after 20 min of ultrasonication. X-ray diffraction (XRD) analysis showed two distinct diffraction peaks at 2θ = 10° and 2θ = 20°, indicating altered crystallite sizes post-ultrasound treatment. Ultrasonication induced structural and conformational changes in the pumpkin seed protein isolates, as confirmed by SDS-PAGE and weight loss analyses. Alterations in the SDS-PAGE profile and reduced weight loss were associated with improved solubility and enhanced thermal and functional properties in the treated pumpkin seed protein isolates. This emphasizes the potential of PSPI to increase their value-added potential through ultrasonication.

## 1 Introduction

The need for protein sources that are cheap, rich in nutrition, possess sufficient functionality, and have all the necessary characteristics to be utilized as functional ingredients in the food industry has grown during the last several years. Utilizing agro-industrial byproducts like proteins for production could be a long-term approach that would raise the byproducts’ market and financial worth. One long-term approach to increase the economic worth of these by-products could be to recover protein from agro-industrial production losses [1]. The pumpkin seed meal, a by-product of pumpkin seed oil production could fulfill such requirements. Pumpkin seeds can be regarded as a beneficial and valuable source of protein [2]. Plants in the *Cucurbitaceae* family, which includes the pumpkin, are mostly grown in Asia and some regions of Europe. The Food and Agriculture Organization of the United Nations research studies state that the production of pumpkins reached around 27 million tons worldwide in 2020, with 63 % originating from Asia, led by China, which produced the most at around 7.5 megatons [3].

According to Ahmad et al. (2019), pumpkin seeds make up 3.1 % of the total weight of the fruit and provide an abundance of protein (33 %), oil (47.3 %), and high concentration of sulfur amino acids with low concentrations of phytic acids as well as trypsin inhibitors [4]. Because pumpkin seeds are an excellent source of unsaturated fatty acids, secondary plant compounds, and protein (24.5 to 36 g/100 g), [5], their popularity for direct consumption is also increasing steadily [6]. The American Heart Association recommends consuming around 30 g of pumpkin seeds daily since they include various nutrients that have been shown to have favorable benefits on bone and heart health [7]. According to Bucko et al. (2016), the protein content of the de-oiled fraction can rise to as high as 65 % following the removal of any remaining oil from the pumpkin press cake [8]. Additionally, the 12S globulin predominates in pumpkin seeds and has structural similarities to globulins in legume seeds [8]. This resemblance suggests that PSPI (Pumpkin-Seed Protein Isolate) exhibits comparable functional characteristics to proteins from legume seeds, including foaming, gelling, and emulsification properties [9].

However, functional qualities that are desired by the food business are not demonstrated by the majority of natural proteins. Many parameters, including temperature, pH, ionic strength, enzymatic activity, and physical and chemical approaches, can change the special functional properties of protein isolates [10], [11]. Since the native structure of protein molecules has limited functional properties, these techniques are used to give the native structure desirable functionality. Since physical protein modification is safe, it is frequently employed to give proteins the desired functional characteristics and customize them for various dietary applications.

Ultrasound is a green technique that holds great promise for the processing of food and the extraction of bioactive compounds from animal, marine, and plant sources. It is low-cost, time-consuming, and has minimal environmental impact [12], [13].Ultrasound comprises of the mechanical waves that exceed the hearing limit of the human ear (~ 20 k Hz). Ultrasound processing of protein isolates can be carried out either in bath or by the probe type method, wherein, the latter has been used widely.According to O'Sullivan et al. (2016), the basis of ultrasonic technology is sound waves having frequencies greater than 20 kHz, which surpasses the threshold of human hearing and it is non-toxic, safe, and environmentally beneficial [14], [15]. Based on frequency ranges, High-intensity ultrasound (low frequency, 20 to 100 kHz) (HIU)and low-intensity ultrasound (high frequency, 100 kHz – 1 MHz) are the 2 categories into which ultrasound can be classified. Low-intensity ultrasound is regarded as a non-destructive ultrasound and is frequently employed for the following purposes: 1) food property measurement; 2) flow rate measurement; and 3) Food package inspection.

However, protein structural alterations have been determined by the use of high-intensity ultrasonic technology, which aims to enhance the solubility and other functional characteristics of proteins [16]. According to reports, sonication-induced structural modifications result in the formation of protein isolates possessing a higher structured composition [17], enhanced gelling characteristics as well as protein isolate gel strength [18]. According to Resendiz-Vazquea et al. (2017), the application of ultrasound treatment caused the protein isolates microstructure to be disturbed, leading to the production of more large aggregates. In an investigation by Zhang et al. (2014) on PPI (Peanut Protein Isolate), ultrasonic treatment changed the protein's globular form to a mesh structure. Ultrasonic treatment was observed to enhance the PPI's emulsifying characteristics. The average particle size dropped from 474.7 nm-255.8 nm, while the molecular weight remained unchanged [19]. According to Wang et al. (2020), there was a notable enhancement in the emulsifying, rheological, and stability characteristics of the chicken MP (Myofibrillar Protein) treated with ultrasound. Ultrasound remarkably decreased the particle size of MP and facilitated the development of smaller, more consistent emulsion droplets [20].

Canola protein isolate's (CPI) structural, functional, and physical–chemical characteristics were altered by ultrasound treatment [21]. Reduced bulk density, moisture content, water activity, and L* & a* color parameters were the outcomes of the ultrasound treatment.The findings of the SDS-PAGE investigation demonstrated that the protein electrophoretic patterns were unchanged, indicating that covalent connections were not disrupted by sonication and according to scanning electron microscopy(SEM), the ultrasound treatment caused the CPI's microstructure to be disturbed, which caused bigger aggregates to develop in the lyophilized powder. In addition, following divergent ultrasonic therapies, the protein structure as well as antioxidant activity of watermelon seed protein (WSP) were altered [22]. The outcomes revealed a substantial effect of pretreatment with slit divergent ultrasound (SDU, 20/28KHz) on the enzymatic efficiency and structure of WSP.

Martinez-Velasco et al. (2018) concluded that HIUS is an effective tool for improving the foaming and surface properties of faba (Vicia faba L.) beans [23]. According to Vargas et al. (2021), HIUS processing enhances the whey protein isolate's ability to emulsify [24]. Zhu et al. (2018) investigated how HIUS affected the physical and chemical characteristics of walnut proteins. According to the reports, sonication enhanced the emulsifying qualities of walnut proteins, reduced the number of big aggregates, and increased the solubility of the proteins in water [25].

The current study was conducted to investigate the effects of HIUS (probe type method with an amplitude of 25 %, power output of 500 W, and time 5, 10, 20, and 30 min, respectively) on the physicochemical, functional, molecular, and thermal properties of pumpkin seed protein isolate in light of the desired functional attributes of HIUS treatment on the protein isolates. In the near future, products made from traditional cereal crops may be replaced by the use of pumpkin seed proteins in a variety of food items, especially in gluten-free and essential amino acid-rich diets.

## 2 Material and methods

### 2.1 Materials

Pumpkin seeds of the variety PAU MAGZ KADDU NO.1,were collected from Punjab Agriculture University located in Ludhiana, Punjab, India. All other chemicals and reagents used in this study were of analytical grade.

### 2.2 Methods

#### 2.2.1 Preparation of defatted pumpkin seed meal

The defatted pumpkin seed meal was prepared following the method of Devi et al. (2019), with slight modifications. Ground pumpkin seed powder was defatted by extracting fats with n-hexane (n-hexane ratio of 30:1, v/w) under continuous stirring at 40 °C for 36 h using a magnetic stirrer. The mixture was then centrifuged at 6000 rpm for 30 min to separate the solid phase, which was dried at 40 °C, ground into a fine powder, and stored at 4 °C until further use [26].

#### 2.2.2 Preparation of pumpkin seed protein isolates (PSPI)

Protein isolates from defatted pumpkin seed meal were prepared using the method followed by Vinayashree et al. (2021) with slight modification. The defatted pumpkin seed cake was suspended in an alkaline solution adjusted to pH 11.0 using 1 M NaOH. After 30 min of gentle stirring, the mixture was filtered, and the dissolved proteins in the filtrate were precipitated by lowering the pH to 4.0 with 1 M HCl. The resulting precipitate was separated by centrifugation at 4 °C and 5000 rpm for 30 min. The sediment was then washed with deionized water until neutral, separated again by centrifugation, and freeze-dried using a lyophilizer (LABFREEZE Scientific Limited).The PSPI (Pumpkin Seed Protein Isolate) was stored at −80 °C for subsequent analysis. Protein content in the PSPI was determined to be 85.78 ± 0.05 % using the Kjeldahl nitrogen method (n = 3) [2].

#### 2.2.3 Ultrasound treatment

Using distilled water, 10 % w/v dispersions of pumpkin seed protein isolate were made, agitated for 2 h, and then stored at 4 °C for overnight. Following that, an ultrasonic processor with a titanium probe (Qaaf Healthcare International, India) was used, protein dispersions were sonicated in an ice-filled beaker while maintaining the temperature below 10 °C. To maintain a constant temperature, ice was added to the bath every five minutes. Protein dispersions were subjected to 20 kHz (500 W and 25 % amplitude) sonication for 5, 10, 20, and 30 min. The samples were then stored for further investigation after being “lyophilized (LABFREEZE Scientific Limited).

### 2.3 Physicochemical characteristics of PSPI

#### 2.3.1 Solubility

The solubility of pumpkin seed protein isolates was assessed as per the technique described by Malik et al. (2017) with slight modifications. The suspension, comprising 2 % protein, was agitated for one hour at room temperature using a magnetic stirrer. Subsequently, after centrifuging the suspension at 3000 rpm for 20 min., the protein content in the supernatant was calculated employing the Kjeldahl technique [27]. After that, solubility was calculated as follows:

$$S\%=\frac{\mathrm{Amount}\;of\;Protein\;in\;supernatant}{\mathrm{Amount}\;of\;Protein\;in\;sample}\times 100$$

#### 2.3.2 Emulsification properties

Using the technique described by Lawal et al. (2007) with slight modification, the EA (Emulsifying Activity) & ES (Emulsion Stability) of pumpkin seed protein isolates had been assessed. 5 mL of soybean oil was employed to homogenize a 2 % protein solution. After centrifuging the resultant emulsion for five minutes at 3500 rpm, the heights of the entire contents in the tube as well as the emulsified layer were measured [28]. The following calculation was employed to determine the EA:

$$EA\;\left(\%\right)=\frac{Height\;\;of\;\;emulsified\;\;layer}{Height\;\;of\;\;total\;\;contents\;\;in\;\;the\;\;tube}\times 100$$

The stability of the emulsion was assessed by subjecting it to a temperature of 80 °C for 30 min., followed by centrifugation at 3500 rpm for 5 min. ES was then quantified as follows:

$$ES\;\left(\%\right)=\frac{Height\;\;of\;\;emulsified\;\;layer}{Height\;\;of\;\;total\;\;contents\;\;in\;\;the\;\;tube}\times 100$$

#### 2.3.3 Color characteristics

The variables L*, +a*, −a*, +b*, and –b* represent lightness, redness, greenness, and blueness, respectively and were calculated using the method described by Salcedo-Chávez et al., (2002).

Using a Hunter Colorimeter fitted with an optical sensor (Model D25, Optical Sensor; Hunter Associates Laboratory Inc., Reston, VA, USA), the color characteristics of the native and ultrasonic treated PSPIs were assessed based on the International Commission on Illumination (CIE) protocol for the calculation of L∗, a∗, and b∗ [29].

#### 2.3.4 Particle size determination

The analysis of particle size for pumpkin seed protein isolates was conducted utilizing a laser light diffraction particle size analyzer (Shimadzu SALD-2300, manufactured by Shimadzu Corporation, Kyoto, Japan). A prepared protein dispersion was added drop by drop into a cuvette until the refractive index dropped between 20 & 40 percent. Percentage of different size particles was determined and average particle size was the particle size of 50 % normalized particle amount [27].

#### 2.3.5 Turbidity

A Shimatzu UV 1203 UV–Vis spectrophotometer (Japan) was employed to measure the turbidity of a dispersion containing 1 % pumpkin seed protein isolates. Absorbance readings were taken at 600 nm under room temperature conditions, serving as an assessment of turbidity [27].

#### 2.3.6 Sodium dodecyl-sulfate polyacrylamide gel electrophoresis (SDS-PAGE)

The SDS-PAGE profile was carried out using the method as stated by Cao et al. (2020). Following the dissolution of PSPI and sonicated PSPI samples with a constant protein concentration (1 mg/mL), 5 × loading” buffer was added. After five minutes of 90 °C incubation, the solutions were poured into the gel slot. 10 % (w/v) was determined to be the separation gel's optimal concentration. Electrophoresis was performed on “the stacking gel and the separating gel at 100 V & 200 V, correspondingly. The gel was first stained for 30 min with 0.1 % Coomassie Brilliant Blue R-250 dye. It was then destained in a solution made of 25 % methanol & 8 % acetic acid until the required amount of color density was obtained. The molecular weights of the proteins were” ascertained by running the protein markers (10–250 kDa) in parallel on the same gel. The gel was then examined and captured on camera and photographed [30].

#### 2.3.7 SEC-HPLC

The size” distribution profiles of PSPI samples subjected to ultrasound treatment were examined using size exclusion chromatography (SEC) using method described by DuPont et al., (2008).

This analysis utilized a tandem column setup comprising a “TSK G 2000 SW column (7.5 mm × 60 cm, 1 μm, TosoHaas, Montgomeryville, PA, USA) connected to a TSK guard column (7.5 mm × 7.5 cm). Prior to injection, the protein samples were diluted to a concentration of 2 mg/mL and” filtered through a 0.45 μm membrane. A 50 μL injection volume was used. Using an HPLC-UV system, elution had been performed using 0.1 M sodium phosphate buffer at a flow rate of 0.3 mL/min. A UV detector was utilized for detection, which was done at 280 nm [31].

#### 2.3.8 Differential scanning calorimetry

The thermal characteristics of PSPIs were assessed using a DSC 214 Polyma instrument from Netzsch, Germany. Samples weighing 10 mg sample were securely sealed in aluminum pans and compared against an empty pan as a reference, while DSC melting profiles were generated following the approach as stated by Sahraee et al. (2017). Under the conditions of nitrogen flowing at 40 mL/min, heating was performed at a rate of 10 °C/min within the temperature range of 20–300 °C. Then, using peak analysis software, the acquired DSC data was examined, and the onset, denatured and conclusion temperatures were noted [32].

#### 2.3.9 Thermal gravimetric analysis (TGA)

The Perklin Elmer, Diamond TG/DTA was employed to determine the thermal gravimetric analysis of pumpkin seed protein isolates as per the method described by Yu et al. (2015). Protein isolates were heated in atmospheric nitrogen (200 mLmin^−1^) at a rate of 10 °C/min between 40 & 750 °C. The” first process was linked to water loss by evaporation, whereas the second step—which corresponded to weight loss—was attributed to the “degrading temperature [33].

#### 2.3.10 Fourier transform infra red (FT-IR) spectroscopy

The infrared spectroscopy of pumpkin seed protein isolates was” done via Cary 630 FTIR, Agilent Technologies, USA. Protein isolates were combined “with moisture-free potassium bromide at a ratio of 1:100 (w/w) to form protein isolate pellets. These” pellets were then compressed using a vacuum hydraulic press and FTIR spectra were “recorded in the range 4000–600 cm^−1^ [34].

#### 2.3.11 Circular Dichrosim (CD)

Employing “a CD spectropolarimeter (Jasco 810, Jasco Corp., Tokyo, Japan) in a 0.1 cm quartz CD cuvette (Hellma, Muellheim, Baden, Germany) at 25 °C, CD spectra in the far-UV region (260 to 180 nm) were obtained using method described by Kim et al. (2004). In order to remove any insoluble residues, freeze-dried PSPIs were first dissolved in 0.01 M sodium phosphate buffer (pH 7.0) and centrifuged at 8000g for 15 min at 25 °C. For” CD analysis, the protein content was maintained constant at 0.1 mg mL^−1^. Set at 100 nm min^−1^, 0.25 s, & 1.0 nm, correspondingly, were the scan rate, reaction time, and bandwidth. The average of the three scans was utilized to create each spectrum. The “Yang-Us, jwr software from Jasco Corp. was employed to estimate the secondary structure of PSPIs. It computed four different forms of secondary structures: α-helix, β-sheet, β-turn, and random coil [35].

#### 2.3.12 2,2 diphenyl-1-picrylhydrazl (DPPH) radical scavenging activity

With a few minor adjustments, the DPPH technique was used to determine the antioxidant capacity in accordance with Gao et al. (2002). Briefly, a solution of DPPH at 100 μM was made using 100 % methanol. An incubation period of 30 min was followed by the addition of 100 μL of DPPH solution to each experimental condition. The plate reader Stat Fax 3200 (Awareness technology INC) was used to measure the absorbance at an optical density of 512 nm. The results were performed in triplicates [36]. A percentage of radical scavenging activity (RSA%) was used to quantify the antioxidant activity using Formula:

$$\%\left(DPPH\right)Activity=\frac{\mathrm{Acontrol}-Asample}{\mathrm{Acontrol}}\times 100$$

A_Blank_- Absorbance of the control sample (methanol 100 % + DPPH in methanol 100 %).

A_Sample_- Absorbance of the sample (experimental samples in methanol 100 % + DPPH in methanol.

#### 2.3.13 X-ray diffraction (XRD)

Protein structural properties were measured utilizing an X-ray diffractometer (D8 Advance, Bruker, Karlsruhe, Germany) with” CuK (A°) = 1.54056 radiations. Stainless sample holders have been employed to press the protein powder. The samples were scanned with a step size of 0.0131° at “a rate of 3.2°/min between 5° and 40° (2θ). Crystallinity metric was performed by Xpert High Score Plus software [37].

#### 2.3.14 Transmission electron microscopy (TEM)

In pH 2.5 DI water, the freeze-dried PSPI samples were diluted 500 times. A TEM (HT7800, “Hitachi High-Technologies Corp., Tokyo, Japan) operating at 100 kV was employed to record the TEM morphologies of the samples [38].

#### 2.3.15 Scanning electron microscopy (SEM)

Scanning electron Microscope (JEOL, JSM-6390LV, Japan) operating at a 20 kV accelerating voltage was used to analyze the surface morphology of the freeze-dried PSPI samples. Utilizing an ion sputter coater, the samples were coated with gold prior to” examination under the SEM [39].

#### 2.3.16 Statistical analysis

All experiments were performed in triplicate, and results were presented as mean values with standard deviations (± SD). Data analysis was conducted using one-way analysis of variance (ANOVA), and significant differences among mean values were identified through Duncan's multiple range test (p ≤ 0.05) using SPSS version 16.0.

## 3 Results & discussion

### 3.1 Physicochemical characteristics of PSPI as affected by ultrasonication

#### 3.1.1 Solubility

Solubility is a key indicator for evaluating protein denaturation and aggregation, effectively predicting protein functionality [40]. Table 1 presents the solubility results for both native and ultrasound-treated pumpkin seed protein isolates (PSPI). The solubility of the ultrasound-treated PSPI was significantly higher (p ≤ 0.05) compared to the native PSPI, with high solubility achieved at 20 min of sonication. This enhancement in solubility from high-intensity ultrasound (HIUS) results from structural modifications in the proteins’ globular shape, which reveal hydrophilic groups and improve electrical conductivity. Liu et al. (2017) noted that HIUS also reduces protein particle size, which increases water solubility by expanding the surface area between protein and water molecules [41]. Similar increases in solubility after ultrasonication have been reported for black bean protein isolates [42], soy protein isolates [40], milk protein concentrates [43], sunflower protein isolates [27], and walnut protein isolates [25]. The rise in protein solubility due to sonication may also result from the breakdown of internal hydrogen and hydrophobic bonds, enabling hydrophilic amino acid groups to engage with the polar environment [44]. Additionally, sonication-induced cavitation reduces protein molecular weight, enhancing interaction between protein and water molecules. However, prolonged sonication (30 min) led to a decrease in solubility, likely due to aggregation of denatured proteins forming high molecular weight complexes that reduce solubility [27].

**Table 1:** Effect of sonication on Emulsifying Activity (EA), Emulsifying stability (ES), Solubility, Turbidity and particle size of pumpkin seed protein isolates (PSPIs).

Time	EA (%)	ES (%)	Solubility (%)	Turbidity	Particle Size (µm)
Control	52.48 ± 0.04^e^	53.55 ± 0.04^e^	16.26 ± 0.03^e^	0.96 ± 0.03^a^	117.46 ± 0.40^a^
5 min	55.54 ± 0.02^d^	62.15 ± 0.02^d^	38.46 ± 0.02^d^	0.81 ± 0.01^b^	106.50 ± 0.40^b^
10 min	61.27 ± 0.02^c^	67.25 ± 0.04^c^	43.43 ± 0.04^c^	0.73 ± 0.02^c^	95.36 ± 0.31^c^
20 min	67.54 ± 0.04^a^	74.85 ± 0.03^a^	48.25 ± 0.05^a^	0.62 ± 0.04^e^	85.26 ± 0.04^e^
30 min	63.66 ± 0.03^b^	71.25 ± 0.02^b^	45.34 ± 0.03^b^	0.66 ± 0.02^d^	91.43 ± 0.04^d^

(n = 3), Results are expressed as mean values ± standard deviations. Means with different superscript in a column differ significantly (p < 0.05).

#### 3.1.2 Emulsification properties

The emulsifying properties of pumpkin seed protein isolates were determined through emulsifying activity (EA) and emulsion stability (ES). EA indicates the protein's ability to adsorb at the water–oil interface, while ES reflects the protein's capacity to remain at the interface after storage or heating of the emulsion. The highest emulsifying capacity was observed in pumpkin seed protein isolates sonicated for 20 min (Table 1). However, extending sonication to 30 min led to a reduction in their emulsifying capacity. This improvement in both EA and ES was due to the unfolding of protein molecules caused by high-intensity ultrasound (HIUS), which facilitated protein diffusion to the air–water interface, enhancing emulsifying properties. Ultrasound treatment also increased the solubility of pumpkin seed protein isolates, allowing more proteins to be available at the oil–water interface during emulsification. The extreme temperatures and pressures generated by ultrasound likely drive these changes in the emulsifying behavior of protein isolates [19]. At longer sonication times, the decline in EA and ES is attributed to aggregation of denatured proteins. According to Soria and Villamiel (2010), denatured proteins expose more hydrophobic groups, often leading to aggregation. Therefore, an optimal balance between hydrophobic group exposure and protein aggregation is necessary [45]. Similar results have been reported for nut proteins [25], egg proteins [46], soy proteins [47], and peanut proteins [19].

#### 3.1.3 Particle size determination

Particle size is important factor in determining the functional properties of proteins. Sonication significantly reduced the particle size of pumpkin seed protein isolates compared to their native form (p ≤ 0.05) (Table 1). This result is consistent with findings by Xiong et al. (2018) for pea protein isolates [48], Zhu et al. (2018) for walnut protein isolates [25], and Jambrak et al. (2009) for soy protein isolate [49]. Specifically, pumpkin seed protein isolates sonicated for 20 min demonstrated a substantial reduction in particle size from 117.46 μm to 85.26 μm, while isolates sonicated for just 5 min decreased from 117.46 μm to 106.50 μm. Proteins typically form soluble aggregates in aqueous solutions, which leads to particle size reduction.The cavitational, turbulent, and shear forces produced by sonication break down larger, insoluble protein aggregates into smaller particles, increasing the protein-water interface and enhancing protein-water interactions [40]. Notably, in this study, sonication for 30 min resulted in an increase in mean particle size, indicating the aggregation of protein isolate particles with prolonged sonication (>20 min). Similarly, Gulseren et al. (2007) found that bovine serum albumin (BSA) solutions exhibited increased particle size with extended sonication (>40 min), indicating the formation of small aggregates [50].

#### 3.1.4 Turbidity

The impact of sonication on the turbidity levels of pumpkin seed protein solution are presented in Table 1. The results indicate that the protein solutions became less turbid following ultrasound treatment. For 20 min of sonication, the lowest turbidity values were recorded. However, as the treatment duration increased, the turbidity of the protein solution also increased. The reduction in turbidity of protein isolates following sonication can be attributed to the breakdown of larger aggregates or particles into smaller ones. Sonication applies high-frequency sound waves, causing cavitation in the liquid medium. This cavitation generates intense local pressures and temperatures, leading to the breakup of protein aggregates and the dispersion of smaller particles throughout the solution. As a result, the overall turbidity decreases because smaller particles scatter less light, resulting in a clearer solution. Martini et al. (2010) observed a reduction in turbidity in whey protein suspension following ultrasonication, attributing it to the disruption of “protein–protein interactions. This disruption prevented the formation of large aggregates, leading to the creation of smaller aggregates and ultimately resulting in a clearer solution [51].This finding is consistent with the results reported by Malik et al. (2017) for sunflower protein isolate, further confirming the impact of ultrasonication on reducing turbidity by disrupting protein interactions [27].

#### 3.1.5 Color characteristics

The color characteristics (L*, a*, b* values) of native and sonicated pumpkin seed protein isolates are summarized in Table 2. Significant differences (p ≤ 0.05) were observed in the L* values between sonicated and native pumpkin seed protein isolates, with L* values increasing following high-intensity ultrasound (HIUS) treatment.A similar trend was seen in the b* values, while a* values showed no significant differences (p ≤ 0.05) between sonicated and native isolates. The enhanced color observed after HIUS treatment may result from changes in pigments caused by cavitation effects during sonication [52]. This color variation in HIUS-treated isolates is closely linked to sonication time [53]. Sonication may positively or negatively impact pigments in food, potentially releasing them from proteins or altering pigment-binding sites, thereby affecting light absorption. The improved color in pumpkin seed protein isolates through HIUS could enhance their visual appeal, making them more attractive to consumers.

**Table 2:** Color characteristics of native and sonicated pumpkin seed protein isolates (PSPIs).

Sample	L*	a*	b*
Control	73.52 ± 0.30^b^	0.37 ± 0.24^a^	17.64 ± 0.23^b^
5 min	75.9 ± 0.85^a^	0.29 ± 0.23^a^	18.9 ± 0.16^a^
10 min	74.9 ± 1.48^a^	0.27 ± 0.10^a^	18.4 ± 0.60^a^
20 min	74.3 ± 0.67^a^	0.27 ± 0.15^a^	18.21 ± 0.03^a^
30 min	74.06 ± 0.12^a^	0.18 ± 0.10^a^	17.9 ± 0.04^a^

(n = 3), Results are expressed as mean values ± standard deviations. Means with different superscript in a column differ significantly (p < 0.05).

### 3.2 Effect of ultrasonication on molecular properties of PSPIs

#### 3.2.1 Sodium dodecyl sulphate polyacrylamide gel electrophoresis (SDS-PAGE)

**Fig. 1:**
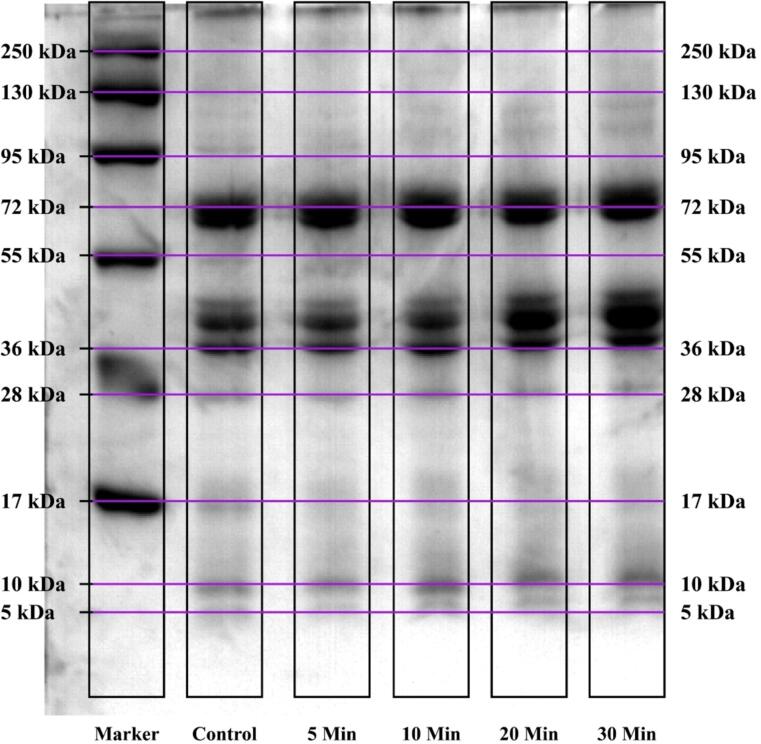
SDS-PAGE profile of native and sonicated PSPIs.

The effect of sonication on the molecular weight of pumpkin seed protein isolates was assessed using SDS-PAGE analysis, as illustrated in Fig. 1. Sonication resulted in significant alterations to the molecular structure of pumpkin seed protein isolates compared to their native form. After sonication, two prominent bands emerged within the 36–72 kDa range across all samples, with the most intense bands observed in pumpkin seed proteins treated for 10 min, followed by those treated for 20, 5, and 30 min. These results are consistent with findings reported by Jambrak et al. (2014) for whey protein isolate [54]. Moreover, a lighter band in the 10–17 kDa range was detected in all sonicated samples, indicating band splitting within the pumpkin seed protein isolates. This splitting is associated with a decrease in molecular weight, which enhances the exposure of protein molecules to water. Similar observations were made by Resendiz-Vazquez et al. (2017) for jackseed protein isolates [16]. The band splitting may result from cavitational forces caused by high shear stress during probe sonication, along with micro-streaming and turbulence effects [42], [47]. The reduction in molecular weight also contributed to an increase in the solubility of sonicated pumpkin seed protein isolates compared to their native isolates, as evidenced by particle size measurements (Table 1), which showed a significant decrease in particle size after sonication. Jambrak et al. (2008) suggested that sonication alters protein conformation, revealing hydrophilic amino acid regions. Furthermore, the changes in the globular protein structure increase the presence of charged groups such as NH_4_^+^ and COO^–^, enhancing protein-water interactions through electrostatic forces, which in turn improves protein solubility [55].

#### 3.2.2 SEC-HPLC

**Fig. 2:**
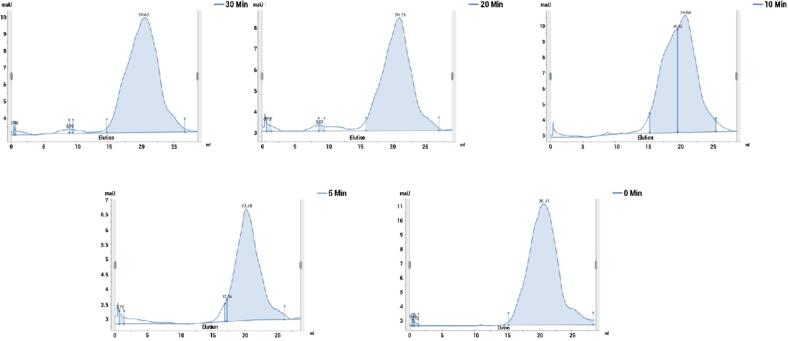
SEC chromatograms of native and sonicated PSPIs.

The molecular weight distributions of PSPIs were analyzed using the SEC-HPLC method. The chromatogram indicates the presence of proteins with varying molecular weight profiles, corresponding to the globulins and albumins present in both native and ultrasound-assisted protein isolates (Fig. 2.). Ultrasound disrupts non-covalent interactions and causes partial unfolding, which can result in fragmentation of protein molecules. Consequently, SEC of ultrasonicated PSPI displays a shift toward smaller molecular weight regions compared to the native form, indicating reduced aggregate size and potentially improved solubility and functional properties, which are valuable in food applications. The peaks observed in the size exclusion chromatography are indicative of globulin fractions, as albumins are not extracted through *iso*-electric precipitation [56].

### 3.3 Effect of ultrasonication on thermal properties of PSPIs

The thermal characteristics, including onset temperature (T_o_), denaturation temperature (T_d_), conclusion temperature (T_c_), and ΔH, for both ultrasound-treated and native PSPIs were evaluated using differential scanning calorimetry and are summarized in Table 3.Among these parameters, T_d_ and ΔH are particularly significant in assessing protein functionality. T_d_ represents the temperature at which protein denaturation begins, whereas ΔH indicates the energy required for the denaturation process to take place.The T_d_ of native as well as ultrasound-treated PSPIs were shown to differ significantly, with sonication lowering PSPIs' denaturation temperature. Specifically, T_d_ decreased with increasing sonication duration, with values of 67.31 °C, 62.71 °C, and 56.38 °C for treatments occurring at 5, 10, and 20 min respectively, showing a corresponding trend in enthalpy of denaturation. This decrease in denaturation temperature may be attributed to structural and conformational changes induced by sonication, causing bond breakage in pumpkin seed protein isolates. However, prolonging treatment up to 30 min resulted in increased T_d_ and ΔH values, reaching 59.81 °C and 39.23 J/g correspondingly. This increase may be attributed to the formation of hydrophobic interactions among disordered protein structures [57]. Comparable outcomes have been noted by Karki et al. (2009) for soy protein isolate [58], Malik et al. (2018) for dephenolized sunflower meal protein isolates [37], and Mir et al. (2019) for HIUS-treated protein isolates from album seeds [59].

**Table 3:** Effect of ultrasonication on denaturation temperature (T_d_) and enthalpy (ΔH) of pumpkin seed protein isolates (PSPIs).

Time	T_o_ (°C)	T_d_ (°C)	T_c_ (°C)	ΔH (J/g)
Control	69.3 ± 0.42^a^	75.6 ± 1.78^a^	104.11 ± 0.75^c^	47.87 ± 1.42^a^
5 min	68.61 ± 1.67^ab^	67.31 ± 1.06^b^	126.09 ± 1.70^b^	45.78 ± 1.74^b^
10 min	66.76 ± 1.20^bc^	62.71 ± 0.79^b^	133.11 ± 2.76^a^	41.25 ± 1.69^c^
20 min	65.4 ± 1.72^c^	56.38 ± 1.69^d^	134.38 ± 1.68^a^	35.43 ± 0.75^e^
30 min	55.54 ± 0.96^d^	59.81 ± 1.19^c^	134.42 ± 1.92^a^	39.23 ± 1.47^d^

(n = 3), Results are expressed as mean values ± standard deviations. Means with different superscript in a column differ significantly (p < 0.05).

### 3.4 Effect of ultrasonication on weight loss properties of PSPIs

**Fig. 3:**
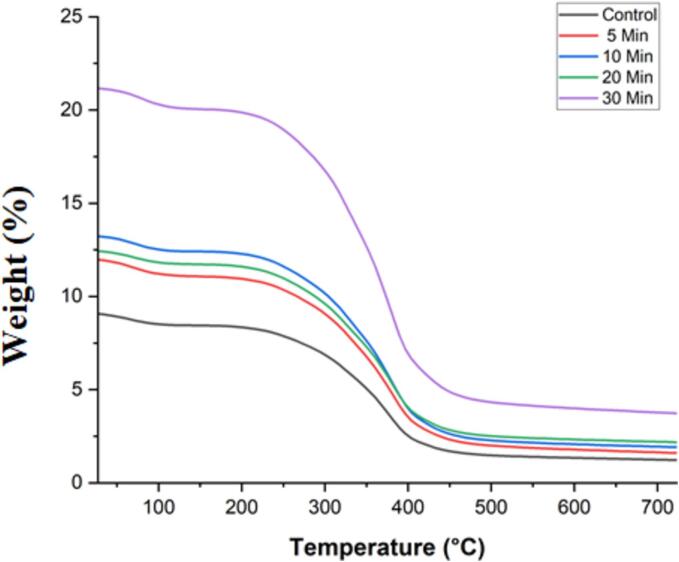
TGA profile of native and sonicated PSPIs.

Thermogravimetric analysis (TGA) was performed on pumpkin seed protein isolates to examine the effects of ultrasound treatment on their thermal degradation behavior.The breakdown patterns of the protein isolates treated with ultrasound and those left untreated were comparable (Fig. 3). A similar trend in weight loss was observed for both native and ultrasound-treated pumpkin seed protein isolates. However, the ultrasound-treated isolates exhibited higher weight loss than the native ones, indicating a reduction in thermal stability following high-intensity ultrasound (HIUS) treatment. This increased weight loss in HIUS-treated isolates is likely due to cavitational and turbulent effects from the ultrasound, which lead to the breakdown of higher molecular weight proteins into lower molecular weight fragments. These smaller protein fragments are more prone to degradation under heat. These findings are consistent with previous research, including studies by Malik et al. (2018) on ultrasound-treated sunflower meal protein isolates [37] and by Mir et al. (2019) on albumin protein isolates [59].The weight loss of native pumpkin seed protein isolates, as well as those treated with ultrasound, progressed through three distinct phases. At first, the weight reduction occurred mainly because the protein molecules were releasing free water and low molecular weight volatiles between 0 and 200 °C. This initial phase was marked by minimal weight loss in pumpkin seed protein isolates, with the extent of weight loss directly correlating with the duration of treatment; longer treatment times resulted in greater weight loss, while shorter durations exhibited the opposite effect. The weight loss was then linked to the breaking of non-covalent bonds, which included electrostatic interactions, hydrophobic contacts, and hydrogen bonds between and within molecules, all occurring between 200 and 400 °C. This phase was characterized by the cleavage of covalent bonds among the amino acid residues [60].Additionally, it was noted that the breakdown of protein isolates intensified with increasing treatment duration, with those treated for 30 min undergoing decomposition more rapidly compared to those treated for 5 min. The final phase of protein breakdown started at 400–700 °C, and it was during this time that oxidation under airflow caused the pumpkin seed protein isolates to lose weight. Prolonged treatment durations during sonication were linked to heightened decomposition rates, with pumpkin seed protein isolates subjected to 30 min of treatment exhibiting significantly accelerated decomposition compared to their native isolates.

### 3.5 Effect of ultrasonication on structural properties of PSPIs

#### 3.5.1 Fourier transform infrared (FT-IR) spectroscopy

**Fig. 4:**
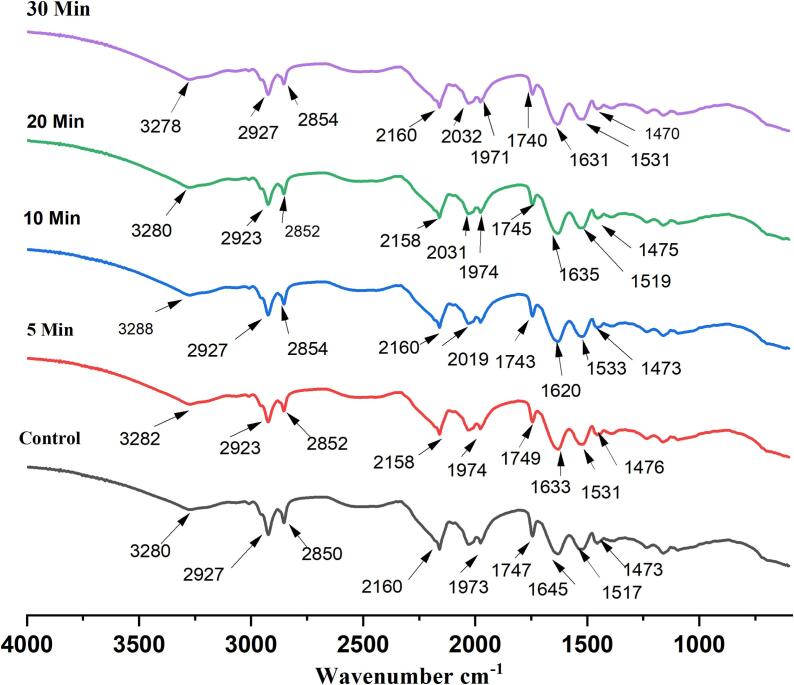
FTIR spectra of native and sonicated PSPIs.

The impact of ultrasonication on the native and sonicated pumpkin seed protein isolate structures was studied employing FTIR, a useful technique for identifying the secondary structure of proteins. Ultrasonication alters the secondary conformation of proteins but promotes peptide formation through bond disruption and fragmentation (Fig. 4). Since amide I is mainly responsive to alterations in protein secondary structure, its spectral positions are primarily associated with the C–O stretching of protein molecules [61]. Different protein isolates have peaks that correlate to the amide I region, which validates the existence of α-helix, β-sheet, β-turn, and random coil structures. The peak of Amide I regions shifted from 1625 cm^−1^ after undergoing ultrasound treatment. In comparison to the native protein isolate, longer sonication times led to lower peak intensities in the amide I region of the protein isolate. This suggests that the ultrasound treatment changed the secondary structure of the protein structure by affecting its vibrational and stretching states. The alteration in secondary structure can be attributed to the disruption of various interactions within protein molecules caused by ultrasonication. These interactions may involve those between neighboring amino acid sequences as well as between different regions of the protein molecule, including disulfide bond [62]. The primary chain of the polypeptide bends and stretches in both directions to produce the prominent peak of amide A, which is located between 3000 & 3500 cm^−1^ [63]. The amide A position shifted in all samples following ultrasonication.Comparable results were noted by Nazari et al. (2018) for millet protein isolate [64] and Badjona et al. (2024) for Faba bean protein isolate [65].

#### 3.5.2 Circular dichroism

Circular dichroism (CD) spectroscopy was used to analyze the secondary structure of PSPI in both native and ultrasound-treated dispersions and the proportions of α-helix, β-sheet, β-turn, and random coil were calculated using Yang's equation [35].Ultrasonic treatment altered the secondary structure of the treated PSPIs, with more significant alterations occurring at longer sonication times. Moreover, samples subjected to 5 and 10 min of sonication exhibited an increase in β-sheet content and a reduction in α-helix and random coil structures compared to the untreated PSPI, as shown in Table 4. On the other hand, PSPI samples subjected to 20 and 30 min of sonication showed an increase in α-helix and random coil content while exhibiting a decrease in β-sheet structures.According to the abovementioned findings, PSPIs' α-helix and random coil structures reduced with shorter sonication times and increased with longer sonication periods. The secondary structure of proteins is influenced by interactions among various molecular components and the local amino acid sequence. Our findings suggest that sonication may disrupt these interactions, leading to changes in the secondary structure of proteins.Chandrapala et al., (2011) found comparable findings, revealing that ultrasound therapy (20KHz, 450 W) boosted the whey protein concentrate's α-helix component and decreased its β-sheet component [57]. Meanwhile, Stathopulos et al. (2004) reported that sonication of proteins at 20 KHz and 30 W showed an increase in β-structure in the aggregates and a concurrent reduction in α-helix structure [17].

**Table 4:** Secondary structural content of native and ultrasonicated PSPIs estimated from CD spectra in the far-UV region (180–260 nm) at 25 °C.

Samples	α- Helix (%)	β- Sheet (%)	β – Turn (%)	Random coil (%)
Control	7.8	62.0	0.0	34.3
5 min	5.2	68.1	3.7	26.1
10 min	6.3	63.5	0.0	28.9
20 min	8.7	57.2	2.3	32.8
30 min	13.0	39.3	9.1	39.4

#### 3.5.3 X-ray diffraction (XRD)

**Fig. 5:**
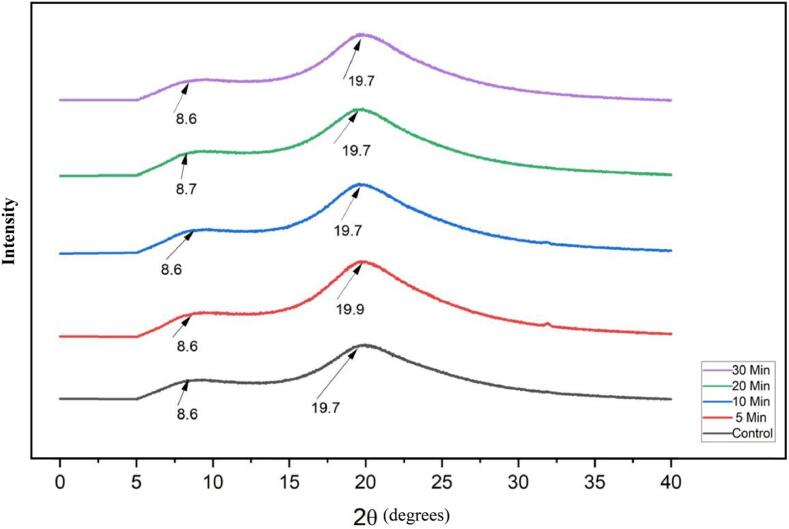
XRD spectra of native and sonicated PSPIs.

The effect of ultrasonication on the crystalline structure of PSPI was analyzed using X-ray diffraction (XRD). According to the XRD spectra, sharp peaks are indicative of crystalline regions, while broad, diffused backgrounds correspond to amorphous regions [66]. XRD patterns for both native and sonicated protein isolate samples exhibited a weak peak around 8°. Furthermore, both untreated and ultrasound-treated PSPI samples revealed a prominent peak at approximately 20°, which suggests the presence of α-helical and β-sheet structures, indicated by distinct peaks at around 2θ = 8.6° and 2θ = 19.7° [67] (Fig. 5).In contrast, commercial soy protein isolate showed multiple peaks between 2θ = 8.90° and 2θ = 25°, likely due to the intensive processing conditions involved in producing protein isolates. The intensity and relative ratio of these peaks in native and treated PSPI, across varying ultrasound treatment parameters, confirmed the differential distribution of α-helix and β-sheet structures. Changes in peak intensity and position after ultrasonication suggest alterations in the crystalline properties of treated PSPI samples [68].Additionally, a clear correlation was observed between peak intensity and particle size, where crystallite size influences both diffraction angle and peak intensity, potentially reflecting conformational changes and structural interactions among proteins [69]. Also,the reduction in crystallite size of ultrasonicated PSPI implies a decrease in particle size [70]. Similar XRD patterns have been reported for other proteins [68], [71]. Consistent with these findings, the current study noted a decrease in crystallinity of pumpkin seed protein isolates following ultrasound treatment, with longer sonication times leading to more pronounced reductions. Initially, the crystallinity of untreated pumpkin seed protein isolates was 24.84 %, which decreased to 21.72 % after 30 min of sonication.

### 3.6 Effect of ultrasonication on antioxidant properties (DPPH radical scavenging activity) of PSPIs

**Fig. 6:**
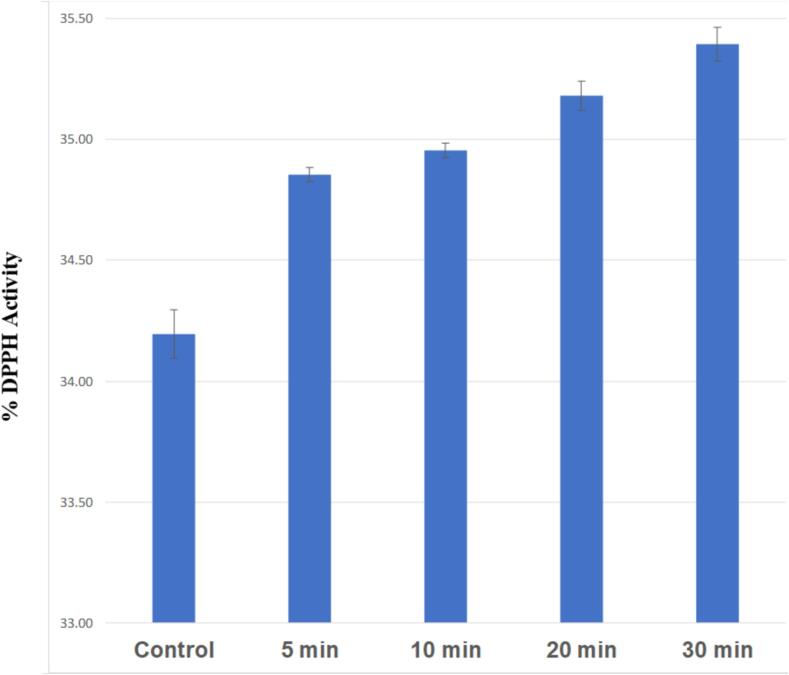
%DPPH activity of control and sonicated PSPIs.

The antioxidant potential of PSPIs was assessed by measuring their DPPH radical scavenging activity (Fig. 6). Results showed notable increases in DPPH scavenging activity in pumpkin seed protein isolates (PSPIs) following ultrasound treatment. Native PSPIs exhibited a DPPH radical scavenging activity of 34.20 %, which increased to 34.85 % (5 min), 34.95 % (10 min), 35.18 % (20 min), and 35.39 % (30 min) post-ultrasound treatment. This enhancement is attributed to the release of low molecular weight peptides during ultrasound cavitation, facilitating their interaction with free radicals. Additionally, ultrasound-induced protein unfolding revealed amino acid residues and side chains with antioxidant properties that were previously concealed, thereby enhancing the overall antioxidant capacity of the PSPIs. [70], [61].

### 3.7 Effect of ultrasonication on morphological properties of PSPIs

#### 3.7.1 Transmission electron microscopy (TEM)

**Fig. 7:**
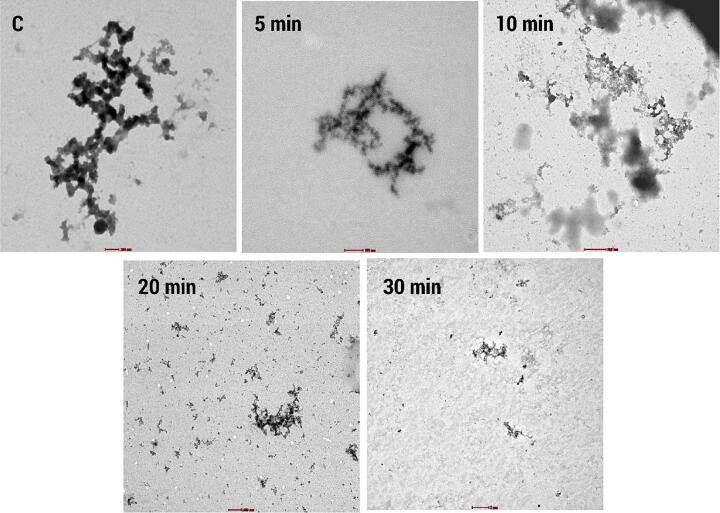
TEM images of native and Sonicated PSPIs.

Ultrasound treated PSPI showed a natural globular protein structure without any linear aggregates, however, ultrasonication can disrupt protein aggregates by breaking intermolecular bonds, leading to the dispersion of individual protein molecules. This might result in a more homogeneous distribution of proteins in the sample, which could be observable in TEM images as a reduction in large, dense protein aggregates. Also, Ultrasonication can alter the size and morphology of protein particles. It may lead to the fragmentation of larger particles into smaller ones or the formation of new structures due to protein unfolding and refolding under the influence of ultrasonic waves. As shown in Fig. 7, TEM images may reveal variations in the size and shape of protein particles when compared to untreated samples [72].

#### 3.7.2 Scanning electron microscopy (SEM)

**Fig. 8:**
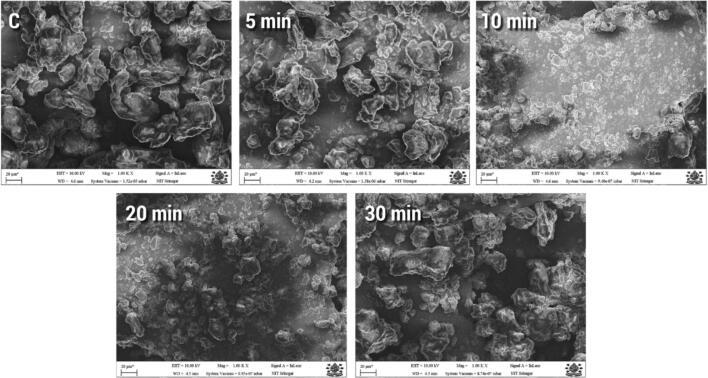
Scanning electron microscopy of native and sonicated PSPIs.

Scanning electron microscopy (SEM) is a widely used technique for analyzing the surface morphology of various biomaterials [73]. The SEM images were obtained at 1000 × magnification. The samples subjected to varying ultrasonic treatment times exhibited loose morphologies with irregular small debris, unlike the dense structure observed in the control (Fig. 8). Additionally, the fragments' dispersion increased as their size and uniformity decreased during the course of the extended ultrasonic processing time. These microstructure changes are most likely caused by the enlargement of embedded hydrophobic and free-SH groups in protein molecules, which causes the molecules to become more visible, [37], [48], hence impacting contact factors like electrostatic effects and surface hydrophobicity [74], leading to irregular fragment formation. These findings align with previous observations on the microstructures of whey protein following HIU treatments [75] and on protein isolates derived from peas [48].Moreover, Jiang et al. (2014) proposed that turbulent forces, micro-streaming, and cavitational force produced during ultrasound treatment could be responsible for alterations in the surface morphology of protein isolates. Protein isolates have been demonstrated to display clumps of different sizes and shapes following ultrasonic treatment, suggesting that the cavitational effect of ultrasound treatment might transform globular structures into mesh structures [37], [19].

## 4 Conclusion

The effects of HIUS on pumpkin seed protein isolates (PSPIs) across varying treatment times revealed significant structural and conformational modifications. The modifications were confirmed using SDS-PAGE, denaturation enthalpy, and particle size analysis. SDS-PAGE results revealed a reduction of high molecular weight protein bands into bands of lower molecular weight, thereby improving the solubility of PSPIs. Additionally, HIUS-treated PSPIs showed reduced denaturation enthalpy and weight loss compared to native PSPIs, confirming structural alterations. Variations in particle size and turbidity further contributed to improved functional attributes, such as solubility and emulsifying capacity. Ultrasonication also significantly boosted the antioxidant activity of PSPIs, as seen in the increased DPPH scavenging activity. The results indicate that HIUS offers a novel, eco-friendly technique for altering the native structure of PSPIs, improving their functional properties for various food applications. The probe-type HIUS modification expands the potential uses of this underutilized plant protein across a wide range of food products. With its high nutritional value and enhanced functionality, this method could facilitate the revalorization of pumpkin seed protein in specialty food production, contributing to waste reduction efforts in India.

## CRediT authorship contribution statement

**Mehvish Habib:** Writing – original draft, Methodology, Investigation, Data curation. **Sakshi Singh:** Writing – original draft, Formal analysis, Data curation. **Sameer Ahmad:** Writing – original draft, Software, Data curation. **Shumaila Jan:** Resources, Methodology, Investigation, Funding acquisition. **Ankit Gupta:** Software, Formal analysis, Data curation. **Kulsum Jan:** Writing – review & editing, Supervision, Formal analysis, Data curation. **Khalid Bashir:** Writing – review & editing, Supervision, Resources, Methodology, Investigation, Conceptualization.

## Declaration of competing interest

The authors declare that they have no known competing financial interests or personal relationships that could have appeared to influence the work reported in this paper.
